# Using Microfluidics to Align Matrix Architecture and Generate Chemokine Gradients Promotes Directional Branching in a Model of Epithelial Morphogenesis

**DOI:** 10.1021/acsbiomaterials.4c00245

**Published:** 2024-07-15

**Authors:** Jessanne Y. Lichtenberg, Corinne E. Leonard, Hazel R. Sterling, Valentina Santos Agreda, Priscilla Y. Hwang

**Affiliations:** † Department of Biomedical Engineering, Virginia Commonwealth University, Richmond, Virginia 23220, United States; ‡ Massey Comprehensive Cancer Center, Virginia Commonwealth University School of Medicine, Richmond, Virginia 23298, United States

**Keywords:** microfluidics, microenvironment, mechanical cues, chemokine gradients, extracellular matrix, fiber alignment

## Abstract

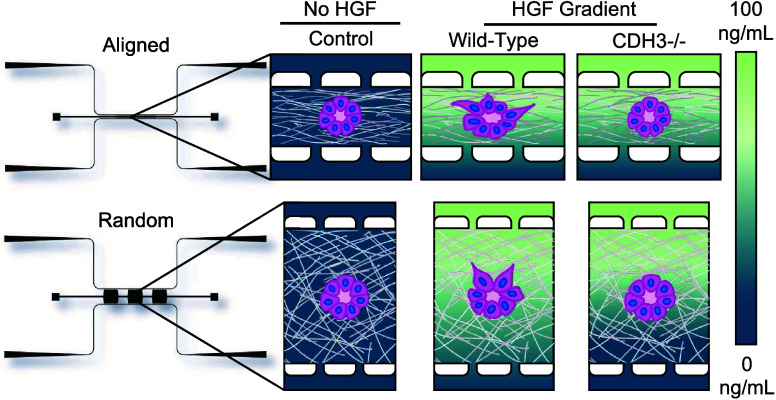

The mechanical cue of fiber alignment plays a key role in the development of various tissues in the body. The ability to study the effect of these stimuli *in vitro* has been limited previously. Here, we present a microfluidic device capable of intrinsically generating aligned fibers using the microchannel geometry. The device also features tunable interstitial fluid flow and the ability to form a morphogen gradient. These aspects allow for the modeling of complex tissues and to differentiate cell response to different stimuli. To demonstrate the abilities of our device, we incorporated luminal epithelial cysts into our device and induced growth factor stimulation. We found the mechanical cue of fiber alignment to play a dominant role in cell elongation and the ability to form protrusions was dependent on cadherin-3. Together, this work serves as a springboard for future potential with these devices to answer questions in developmental biology and complex diseases such as cancers.

## 1 Introduction

Organized extracellular matrix architecture, in particular fiber alignment, is an important and essential cue for promoting cell and tissue development processes.^1−3^ For example, fiber alignment promotes directional cell migration in angiogenesis,^4^ osteogenesis,^5^ and morphogenesis.^6^ Many tissues in the body require organized networks of matrix proteins, including collagen, to produce a favorable environment, such as cardiac and musculoskeletal soft tissues (*i.e.*, ligaments, menisci, or intervertebral disk annulus fibrosis).^7−9^ Moreover, there are diseases where increased remodeling of the extracellular matrix architecture results in aligned fibers, which promotes disease progression, *i.e.*, cancer progression^10−13^ or lung fibrosis.^14−16^ Thus, matrix architecture has a multifunctional role, and there is a need to generate *in vitro* models whereby we can modulate matrix architecture to study biological processes and disease progression.

*In vitro* models to generate aligned fibers have been developed over the years using various approaches, including electrospinning, magnetic fields, or bioprinting.^17−19^ While useful, the scales by which the fibers are produced are on the millimeter scale. Studies demonstrate that millimeter-scale materials do impact cell function, including migration, differentiation, and proliferation.^20^ However, cells also sense at the nano- and microscale level,^21,22^ including mechanotransduction events or cell adhesion-dependent signaling. Further, being able to perform studies at the nano- and microscale allows us to systematically investigate individual cell response and functions, which we may not be able to resolve at a millimeter-scale level.^20^ Here, we present a microfluidic device where we can precisely control fiber orientation on the submillimeter scale. Our model is on the order of microns, so we can study cell-matrix interactions with a finer resolution to understand the contributions of microarchitecture on cell response.^21^ An advantage of our model is that it leverages intrinsic fiber alignment without reliance on external equipment or manipulation. Further, our model incorporates dynamic fluid flow capabilities to mimic constant interstitial fluid flow experienced by tissues *in vivo.*(23) Finally, our model allows for the incorporation of an extracellular morphogen gradient across the tissue chambers in a perpendicular direction to fiber alignment so we can induce two different cues simultaneously in different directions to understand the contributions of each cue separately.

To demonstrate the biological relevance of our microfluidic system, we used an *in vitro* model of epithelial morphogenesis,^24^ where epithelial cells form cysts (also known as acini or spheroids) when embedded in a three-dimensional (3D) matrix. Similar to simple epithelial tissues, these cysts form a lumen with apical-basolateral polarity and develop branching tubules when exposed to hepatocyte growth factor (HGF).^24−26^ A lot of work has been done to understand how microenvironmental cues affect cyst development and morphogenesis. For example, studies demonstrate certain extracellular matrix ligands, such as collagen 1 and laminins, contribute to successful lumen formation.^27^ Other studies demonstrate external and internal pressures can impact lumen and cyst size, as well as cell–cell junctions that are necessary for maintaining cyst homeostasis.^28,29^ When considering branching, most of our understanding is limited to the effects of chemical factors in the microenvironment.^30−33^ However, chemical factors are not the only microenvironment cues experienced *in vivo* during epithelial morphogenesis and branching. Thus, in this study, we use our novel microfluidic models where we can modulate matrix architecture to investigate how fiber alignment affects cell protrusions and eventual branching after successful cyst formation.

In this study, we performed theoretical and experimental studies that demonstrate the ability to generate fluid velocities comparable to *in vivo* values, maintain morphogen gradients, and generate different matrix architectures in our novel microfluidic models. Additionally, we demonstrated the biological relevance of our microfluidic models using an epithelial model of branching. Our results demonstrate that in the presence of both a morphogen gradient and mechanical matrix cue, epithelial cysts respond to the morphogen gradient with cell ruffling followed by protrusions in the direction of morphogen gradient within 8 h of gradient exposure. After longer term exposure to the gradient, cysts begin to elongate in the direction of matrix architecture, suggesting that matrix architecture can be used as a pattern to guide directional morphogenesis. Finally, we demonstrate that knocking out cadherin-3 (CDH3) leads to the loss of cell ruffling and protrusions in response to HGF induced morphogenesis. Our microfluidic platform will enable new studies to understand how chemokine gradients and matrix architecture are interlinked to affect microscale interactions between cells and their environment.

## 2 Materials and Methods

### 2.1 Microfluidic Device Design and Fabrication

**Figure 1:**
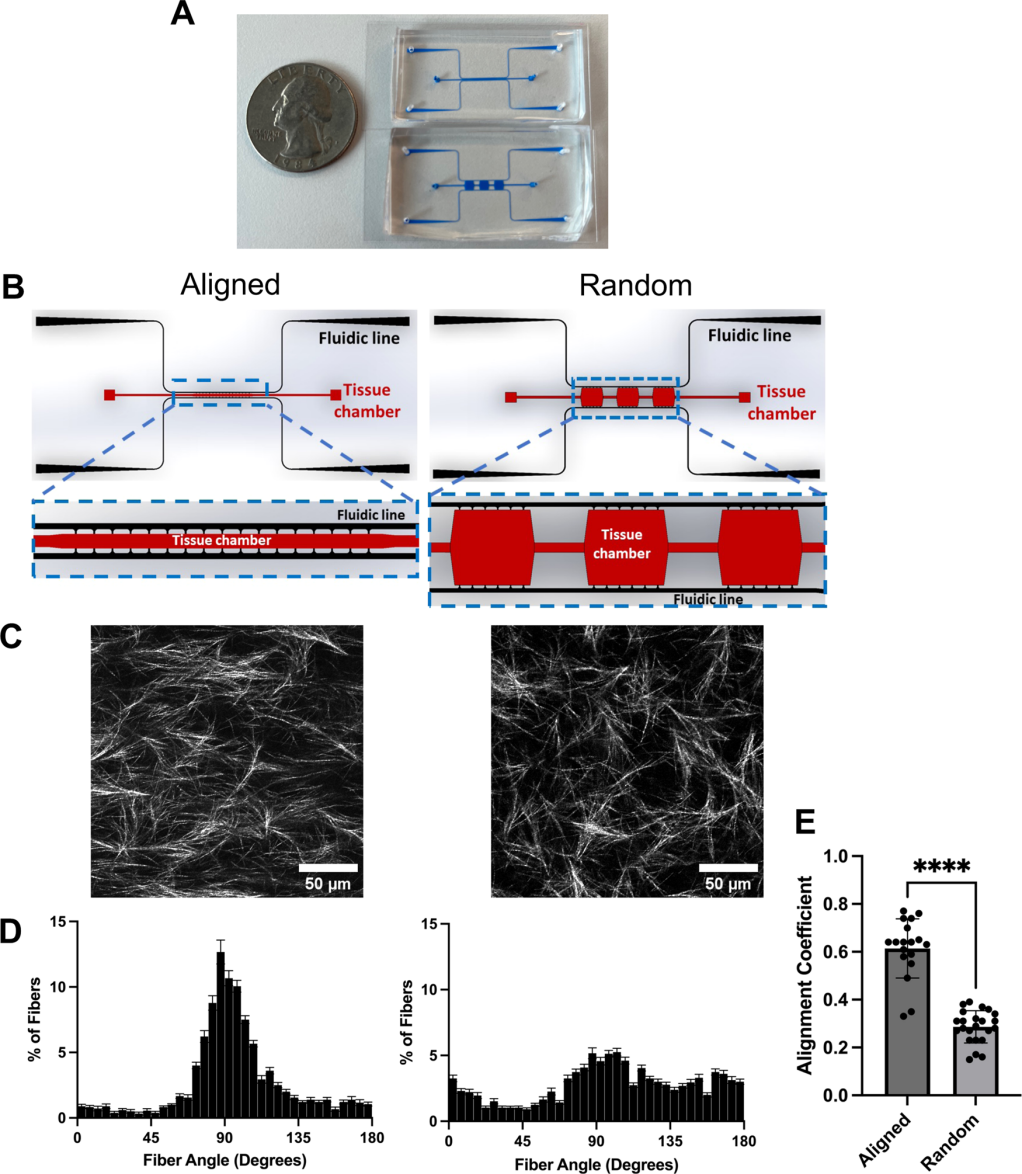
Microfluidic device design for microchannels to produce aligned and randomly oriented collagen. (A) Microfluidic devices made of PDMS and filled with colored dye to highlight the microfluidic channels. (B) CAD models of microfluidic devices with tissue chambers (red) and fluidic lines (black). A close-up model of tissue chambers highlighted in blue box (SolidWorks). (C) Representative confocal reflectance images of collagen organization in microfluidic devices. (D) Histogram of fiber angle frequency in devices. (E) Quantification of fiber orientation using alignment coefficient. *n* = 7–8 devices from 3 to 4 independent replicates. ****p < 0.0001 with student’s *t* test. Data are shown as mean ± SEM.

To generate aligned and randomly oriented collagen type I fiber matrices, two dynamic devices were designed by altering the geometry of the tissue chamber in each device. Previous microphysiological devices containing channels with a 20:1 length/width ratio have observed collagen fiber alignment. Therefore, the microchannel in the aligned device was designed with a length of 5 mm and a width of 250 μm (Figure 1B). The random collagen fiber generating device is used primarily as a control and features tissue channel geometry with a 1:1 length/width ratio (Figure 1B). Fluidic lines run adjacent to each side of the tissue chambers to feed culturing cells with media and establish hydrostatic pressure gradients *via* altering the fluid levels in designated inlet and outlet reservoirs. The fluidic lines are connected to the tissue chambers *via* evenly spaced micropores that also aid in generating even velocities across the porous tissue chamber. The devices were designed in SolidWorks.

Standard photolithography methods were used to generate 100 μm tall molds on silicon wafers for each device using an SU-8 2075. The molds were fabricated in a clean room (Virginia Microelectronics Center, Richmond, VA). The molds were silanized using Trichloro(1*H*,1*H*,2*H*,2*H*-perfluorooctyl)silane to increase hydrophobicity, allowing for multiple uses and easy release of PDMS from the mold. The microfluidic devices were then fabricated by pouring degassed poly(dimethylsiloxane) (PDMS), which is prepared by mixing Dow Sylgard 184 silicone elastomer base and a curing agent with a 10:1 ratio, onto the molds. The PDMS was cured at 70 °C for 3 h and then cut out and peeled from the mold. The device was then bonded to either a glass slide (Corning Plain Microscope slide, 75 × 50 mm^2^) or a cover glass (VWR Micro Cover Glasses, 24 × 50 mm^2^) using a plasma cleaner and cured at 110 °C for at least 10 min. Devices were sterilized *via* an autoclave or ultraviolet (UV) treatment prior to use in experiments with cells.

### 2.2 Computational Modeling of Microfluidic Devices

The SolidWorks CAD models were imported into COMSOL Multiphysics to predict tissue chamber interstitial fluid velocities and the time to chemical gradient formation. Modules include the brinkman equations, utilizing Darcian flow to model the porous medium, and transport of diluted species for modeling chemical gradient formation.^34^ The fluid flow was driven by the hydrostatic pressure heads in the inlets and outlets. The no-slip boundary condition was used. The physical constants used include diffusion coefficient of 70 kDa dextran, 7 × 10^–11^ m^2^/s;^34^ porosity and hydraulic permeability of collagen, 0.99 and 2 × 10^–13^ m^2^;^35^ dynamic viscosity of water, 1 cP; density of water, 1000 kg/m^3^.

### 2.3 Collagen Characterization

To validate that the devices could generate the desired collagen fiber orientation, rat tail collagen type I at a concentration of 2 mg/mL (Corning 354249), with no cells, was loaded into the tissue chambers. The devices were polymerized for 15 min at room temperature and then 1 h at 37 °C. DPBS was added to the fluidic lines to keep the hydrogels hydrated, and they were stored at 37 °C until imaging. Images of the collagen fibers were taken using confocal microscopy (Zeiss LSM 980, 20× or 40× W). Images were processed in Fiji ImageJ to adjust brightness and contrast and were converted to TIF files for processing. Fiber orientation quantification was done using CurveAlign (V5.0 β).^36^ The alignment coefficient value and histogram of fiber angle distribution were used to quantify the difference between aligned and randomly oriented collagen fibers, where a higher value indicates more aligned fibers and a lower value indicates more randomly distributed fibers.

### 2.4 Interstitial Fluid Velocity

Fluorescence recovery after photobleaching (FRAP) was performed on the devices to measure the interstitial fluid velocity within the porous tissue chamber. After the devices were loaded with 2 mg/mL concentration of collagen I (Corning 354249), a 1:10× dilution of 70 kDa FITC-dextran (1.5 mg/mL, Sigma 46945) was added into the fluidic lines to flood the devices. Before experimentation, all fluids in the inlets and outlets were leveled with each other, creating a pressure difference of 0. Immediately before performing FRAP, predetermined volumes were added to each of the inlets and outlets to generate pressure gradients ranging from 2 to 20 mm H_2_O across the tissue chamber. Using confocal microscopy (Carl Zeiss LSM 980), a circular region of 30 μm in diameter was bleached in the center of the microchannel, and images were taken every 0.5 s for a total time of 15 s. Images were cropped, thresholded, and despeckled in ImageJ and then input into a custom MATLAB code to track the centroid of the bleached spot over time to calculate the convective velocity of fluid flow within the device.

### 2.5 Morphogen Gradient Experiments

To confirm the formation of a morphogen gradient by diffusion in each microfluidic device, gradient testing was performed for a total of 24 h. Devices were loaded with 2 mg/mL concentration of collagen I (Corning 354249), loaded with 1× PBS after polymerization, and left to establish flow overnight at 37 °C. Before experimentation, most of the fluid was removed from inlets and outlets, ensuring all fluid heights were leveled. Devices are then loaded with 200 μL of 1:10× dilution of 70 kDa FITC-dextran (1.5 mg/mL, Sigma 46945) in the top inlet and 200 μL of 1× PBS in the bottom inlet. Devices are then placed immediately on the immunofluorescence microscope (Nikon Ti2) and imaged every 30 min for 24 h on brightfield and GFP channels under conditions of 18% O_2_ and 37 °C. Aligned devices were imaged using 10×, and random devices were imaged using 4×. Images were cropped in ImageJ, and the gradient profile was measured across the tissue chambers at time points suggested by the computational modeling (aligned: 0, 3, and 24 h; random: 0, 17, and 24 h).

### 2.6 Generation of CDH3 Knockout Cell Line

MDCK II cells were gifted by Dr. Daniel E. Conway. MDCK cells were cultured in 1X DMEM with 10% Fetal Bovine Serum and 1% Penicillin–Streptomycin at 37 °C. The CDH3^–/–^ cell line was generated using U6-gRNA/CMV-Cas9-GFP (Sigma-Aldrich). MDCK cells were transfected using lipofectamine 2000, and since the CRISPR backbone has a GFP tag, FACs sorting was performed to isolate successfully transfected cells. Isolated cells were grown to confluency, and successful CDH3 knockout was verified *via* Western blot and immunofluorescence staining (Supporting Figure 5). For the Western blot, lysate was collected from 1 million cells using RIPA lysis buffer (Sigma 20–188) and Pierce protease and phosphatase inhibitor mini tablets (Thermo Scientific). Lysate was centrifuged at 15,000 rpm at 4 °C for 10 min, and the cell debris was removed. A 1:1 ratio of 2× Laemmli buffer (Bio-Rad 1610747) with β-mercaptoethanol was added before boiling at 95 °C for 10 min. Protein concentration was measured using a Bradford reagent on a Nanodrop Spectrophotometer (Thermo Scientific). A protein concentration of 69 μg was loaded into 4–20% Mini-PROTEAN TGX Precast Protein Gels (Bio-Rad). Proteins were transferred to a membrane and blocked in 5% BSA for 1 h at room temperature. Primary antibodies in 5% BSA were used to stain blots overnight on the shaker at 4 °C. Secondary antibodies in PBS were used on blots on a shaker at room temperature for 1 h. Primary antibodies include CDH3 monoclonal antibody (1:500×; Invitrogen #32–4000) and α-tubulin (1:1000×; Cell Signaling Technology #2144). Secondary antibodies include anti-rabbit IgG HRP-linked antibody (1:5000×; Cell Signaling Technology #7074) and anti-mouse IgG HRP-linked antibody #7076 (1:5000; Cell Signaling Technology). For verification by immunofluorescence staining, glass bottom dishes (35 mm diameter with 20 mm window) were coated with fibronectin (20 μg/mL). A million cells were seeded into the dishes and incubated for 24 h at 37 °C. Samples were fixed in 4% PFA and washed in PBST. Primary antibodies include CDH3 monoclonal antibody (1:500×; Invitrogen #32–4000) and cadherin-1 (E-cadherin) monoclonal antibody (1:500×; Cell Signaling Technologies #3195S). Species matched Alexa Fluor secondary antibodies were used. Samples were imaged on a Zeiss LSM 980 or Nikon Ti2.

### 2.7 Microfluidic Device Assay

To make 3D MDCK cysts, 45 μL of Matrigel (Corning CB-40230) was plated into the bottom of 8-well chamber slides (Cellvis) so that more Matrigel was in the center of the wells and the bed of gel tapered toward the edges.^29^ After the Matrigel polymerized (20 min at 37 °C), 7500 MDCK cells in media (DMEM 1X supplemented with 10% HI FBS and 1% Penicillin–streptomycin) with 2% Matrigel were added to each well. After 3 days in culture, lumens began to form in the cysts, as confirmed visually. After 4 days in culture, MDCK cysts were extracted from the Matrigel using Corning Cell Recovery Solution.^37^ MDCK cysts were loaded into the devices in a rat tail collagen 1 (2 mg/mL, Corning 354249) hydrogel and polymerized for 15 min at room temperature and then 1 h at 37 °C. Media was added to both fluidic lines, and the devices were cultured at 37 °C. On the following day, media with HGF (50 ng/mL) was added into both fluidic lines (bath) or just the top fluidic line (gradient). HGF at concentrations of 60 or 100 ng/mL was added to the top fluidic lines to generate different mean concentrations and gradient magnitudes (slope). The cells were cultured in HGF overnight (or medium for the no HGF condition) and imaged the following day. After 48 h in culture, tissues were fixed in 10% paraformaldehyde for 24 h, washed in PBST (1× PBS with 0.1% Tween20) for 24 h, and blocked in Abdil (1× PBS with 0.1% Tween20 and 2% BSA) for 48 h. Primary antibodies were incubated for 48 h and included cadherin-3 (Invitrogen 32–4000 at 1:500×). After PBST wash for 24 h, the secondary antibodies were incubated for 48 h (Alexa Fluor secondaries and Invitrogen Phalloidin). DAPI or Hoechst was used for the nuclear counterstain. Microfluidic devices were imaged on a Nikon Ti2 (20×) or Zeiss LSM 980 (40× W).

As a primary cell source, tumor organoids were created from the primary tumors of a spontaneous breast cancer mouse model (MMTV-PyMT). The tumor cells from this model are established to migrate collectively.^38−41^ The mammary gland tumors were isolated from female 12-week-old MMTV-PyMT; K14-GFP mice gifted by the Longmore lab,^39,42^ following the approved IACUC protocol (AD10002197). Freshly isolated tumors were minced and digested in low concentrations of collagenase and trypsin, as previously published.^39^ Tumor organoids were separated out by differential centrifugation and filtered to be between 40 and 100 μm. Tumor organoids were used immediately for all experiments and cultured in standard culture media (DMEM 1X supplemented with 10% HI FBS and 1% Penicillin–streptomycin). Tumor organoids were loaded into the microfluidic devices as described in the methods for the MDCK cysts and cultured for either 24 or 48 h. At the experimental end point, tissues were fixed and stained in the same manner as described earlier for phalloidin and DAPI and imaged on a Zeiss LSM 980 (40× W).

### 2.8 Cell Cluster Characterization

Z-projections were created from phalloidin IF staining images using FIJI ImageJ. From Z-projections, binary images were generated of the cysts and FIJI ImageJ was used to calculate area, perimeter, aspect ratio, roundness, and overall cyst angle. Further, a custom-generated MATLAB code calculated protrusion angles after the user selects individual protrusions within a cell cluster. The MATLAB code calculated the protrusion angle by measuring the angles of each protrusion from the center of each cell cluster centroid normalized to a horizontal axis. Our custom-generated MATLAB code is provided in Supporting Files. Histogram values of the protrusion angles were determined by calculating the frequency of the protrusion angles in 10° intervals in Microsoft Excel using the Frequency function.

Cyst curvature, a parameter to measure how much the perimeter of the cysts deviates from a straight line (*i.e.*, a higher curvature value demonstrates more ruffles or protrusions as the curve will not be straight), was also measured. To understand if chemokine gradients promote directionality, curvature was quantified for the top (closest to the gradient) and bottom half of the cysts separately. First, the centroid of each cyst was identified via FIJI ImageJ and each cyst was cropped to create the top and bottom half. Second, the FIJI plugin, “Kappa-Curvature Analysis” was used to calculate the overall curvature of each half of the cluster.^43^ Finally, a curvature ratio between the top and bottom half of each cyst was calculated to understand differences between the side of the cyst exposed to the larger concentration of the gradient (*i.e.*, top) and the side of the cyst exposed to the smaller concentration of the gradient (*i.e.*, bottom).

### 2.9 Statistical Analysis

For our cell line experiments, we analyzed a minimum of 10 cysts per condition from at least three different replicates. For animal experiments, a minimum of 10 organoids were analyzed from at least three different mice, usually 4–10 organoids per mouse. Only female mice were used since about 99% of breast cancer cases occur in females.^44^ This is based on power analysis: assuming a 50% change between experimental and control groups, a 0.05 significance level, and 0.80 power, it is estimated that at least 10 organoids per experimental condition are required for statistical power. Statistical analysis was performed in GraphPad Prism. Student’s *t*-test or analysis of variance (ANOVA) with Tukey’s post-hoc analysis was performed for all studies. A *p*-value less than 0.05 was considered statistically significant.

## 3 Results

### 3.1 Microfluidic Devices Generate Aligned Matrix Fibers

We created a set of microfluidic devices where we can modulate fiber alignment by changing the tissue chamber geometry. Other studies have demonstrated microchannel geometry can be used to manipulate fiber orientation.^45,46^ To generate aligned fibers, we designed microchannels with a high length to width ratio (aligned devices) (Figure 1; Supporting Figures 1–3). We also created a device to generate randomly oriented fibers by creating microchannels with a 1:1 length-to-width ratio (random device) (Figure 1A,B). To validate we could generate these two different matrix architectures, we loaded each device with 2 mg/mL collagen 1 and let the collagen polymerize. After polymerization, we performed confocal reflectance microscopy to visualize fibers and quantify fiber orientation (Figure 1C; Supporting Figure 1). Aligned devices generated matrix fibers with a mean alignment coefficient of 0.61 compared to 0.29 in random devices (Figure 1D,E).

### 3.2 Microfluidic Devices Generate Dynamic Fluid Flow Velocities and Morphogen Gradients

**Figure 2:**
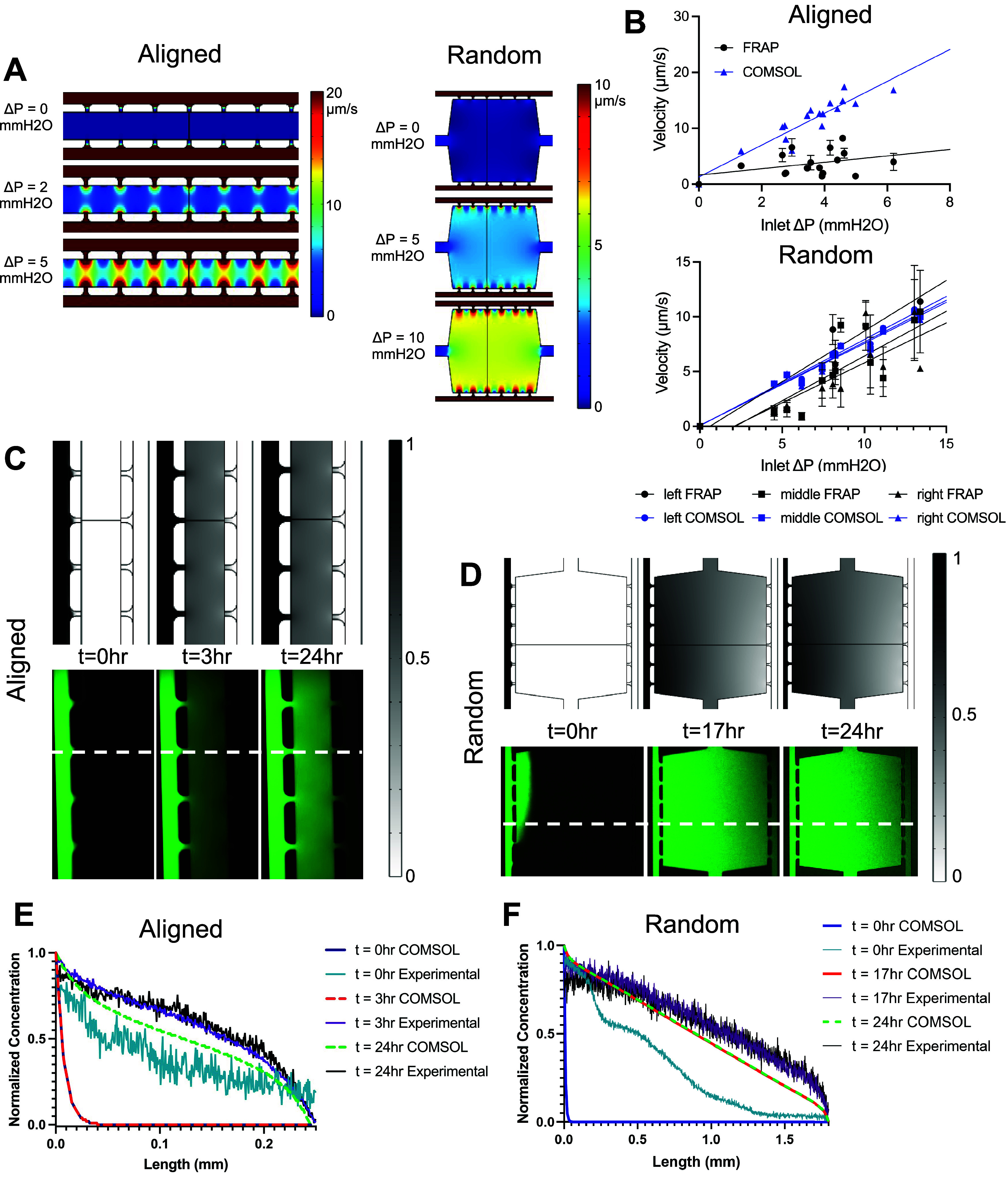
Evaluation of interstitial fluid velocities and morphogen gradient formation in microfluidic devices. (A) Computational modeling of various velocities achieved in devices due to hydrostatic pressure differences in the fluidic lines. (B) Fluid velocities from FRAP experiments (*n* = 7–9 devices from 4–5 independent replicates) compared to computational modeling. (C, D) Modeling of morphogen gradient formation and maintenance in computational (top, gray) and experimental platforms (bottom, green). (E, F) Comparison of computational and experimental gradients (*n* = 3 devices per condition).

*In vivo*, tissues and cells constantly experience interstitial fluid flows. Thus, we wanted to incorporate low levels of fluid flow at all times in our microfluidic design. To accomplish this, the tissue chambers are connected to a pair of fluidic lines *via* micropores for media delivery *via* hydrostatic pressure heads. By altering the differential hydrostatic pressure heads (Δ*P*) from the inlet and outlets, we can tune the interstitial fluid velocity in our devices. We performed computational modeling to identify the generated fluid velocity from a range of Δ*P* values (Figure 2A,B; Supporting Figures 2 and 3). The aligned devices generate fluid velocities from 0 to 20 μm/s, and the random devices generate fluid velocities between 0 and 10 μm/s, depending on the pressure gradient delivered in the fluidic lines; all of the velocity values we measured are within the physiological range.^47^ We also measured the Reynolds number throughout the devices to ensure all regions of the devices are under laminar flow (Supporting Figures 2 and 3).

Further, *in vivo*, tissues contain various extracellular gradients to deliver growth factors or morphogens that can promote cell migration, proliferation, inflammation, or differentiation.^48,49^ In our device, we can generate extracellular gradients through adding a morphogen concentration to one fluidic line inlet (Δ*P* = 0). We performed computational modeling to determine how long it would take an extracellular gradient to form and if it could be maintained up to 24 h. Then, we validated our computational results through the experimental delivery of 70 kDa FITC-dextran as a model morphogen. Computational modeling and experimental validation demonstrate that in the aligned devices, a morphogen gradient is formed in 3 h, while in the random devices, a gradient is formed in 17 h. In both the devices, the gradients can be maintained for 24 h (Figure 2C–F and Supporting Figure 4).

### 3.3 Directional Branching within the Aligned and Random Devices

**Figure 3:**
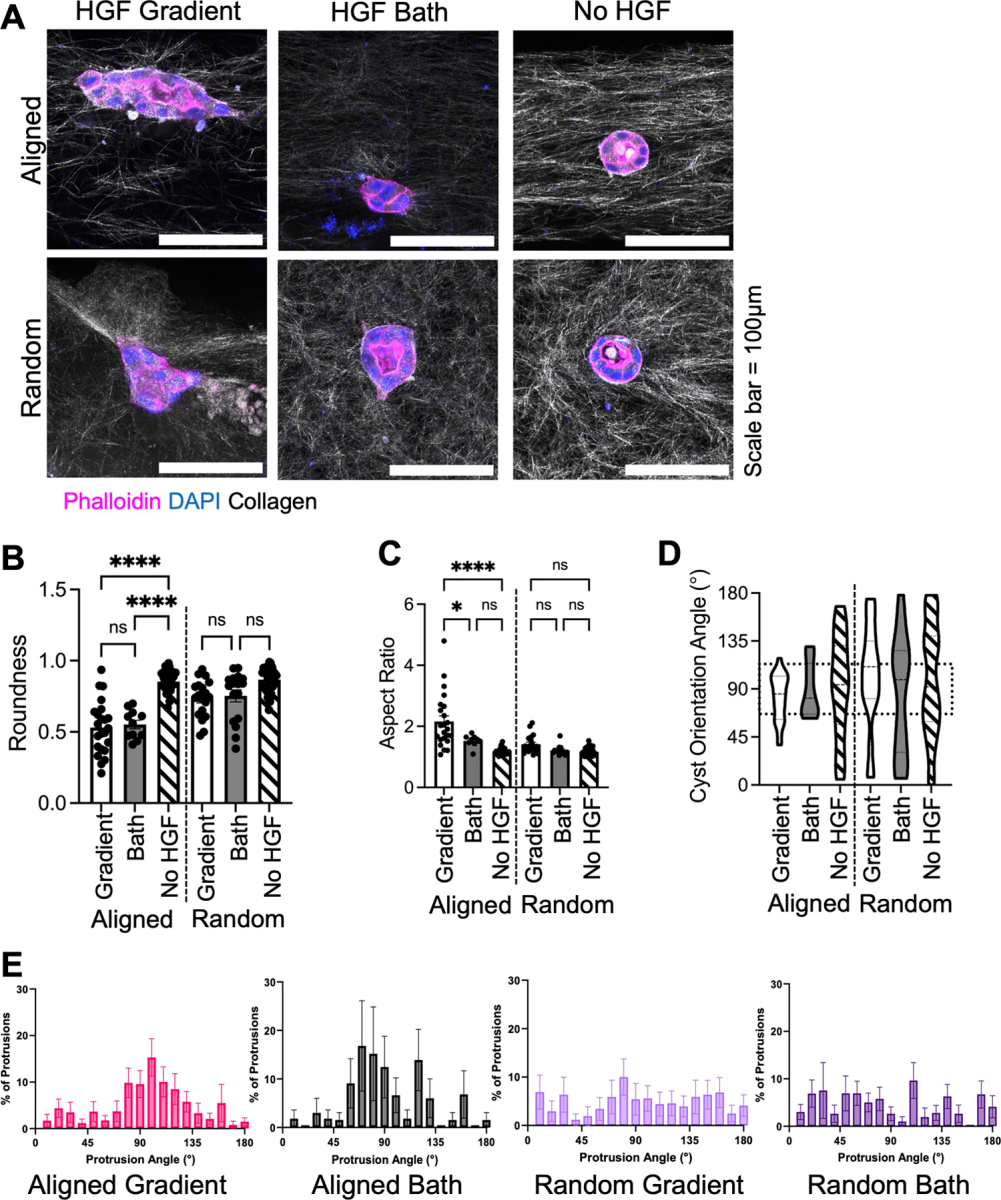
MDCK cysts elongate and form protrusions in the direction of fiber alignment under HGF treatment. (A) Representative immunofluorescent images of MDCK cysts in microfluidic devices under HGF gradient (50 ng/mL) and bath (phalloidin = pink, DAPI = blue, collagen = white, and scale bar = 100 μm). Cyst morphology: (B) roundness, (C) aspect ratio, (D) overall cyst orientation angle, and (E) percent of protrusions orienting in angles 0–180°. All data are presented as mean ± SEM *n* = 3 replicates (11–38 cysts). For all experiments: ns = not significant, *p < 0.05, **p < 0.01, ***p < 0.001, and ****p < 0.0001; ANOVA with Tukey’s posthoc analysis.

We used an epithelial morphogenesis model to demonstrate the biological relevance of our microfluidic devices. After creating MDCK cysts, we encapsulated them in collagen 1 and delivered them into our aligned and random devices. We then added HGF, an established morphogen factor that promotes MDCK cell protrusions,^50^ either as a gradient or bath (50 ng/μL) overnight and characterized changes in cell cluster shape and protrusions (Figure 3A). In aligned devices, regardless of the HGF gradient or bath exposure, MDCK cysts became less rounded (Figure 3B). There were no significant differences in cyst roundness when cultured in random devices, regardless of HGF conditions (Figure 3B). Aspect ratio, a measure of cyst elongation, was significantly larger for cysts cultured in an HGF gradient compared to bath conditions in the aligned devices but not in the random devices (Figure 3C). To understand what direction the cysts elongated, we measured the overall cyst orientation angle; the majority of cysts cultured in aligned devices orient between 70 and 110°, which is the same orientation of the collagen fibers (Figure 3D). We further analyzed the distribution of protrusions, and in aligned devices, the majority of cyst protrusions also orient in the direction of aligned fibers (70 to 110°) in both gradient and bath conditions (Figure 3E). In random devices, cysts formed protrusions between 0 and 180°, indicating they do not form biased protrusions (Figure 3E).

To further validate the biological relevance of our microfluidic devices, we encapsulated primary tumor spheroids in aligned and random devices (Supporting Figure 6). In our prior published work, we demonstrated the ability to promote directional cell migration in response to fiber orientation in a static assay.^50^ Here, we asked whether the addition of dynamic interstitial fluid flow affects tumor organoid sensitivity to mechanical matrix cues and whether these sensitivities will affect collective migration. Thus, we cultured primary tumor organoids (MMTV-PyMT) in aligned and random devices under low interstitial fluid flow (∼3 μm/s). First, we quantified changes in collagen matrix properties (Supporting Figure 6A; confocal reflectance imaging). We generated a histogram of collagen fiber orientation in aligned and random devices after 24 and 48 h of culture in low fluid flow (Supporting Figure 6B). In aligned devices, the majority of collagen fibers orient between 70 and 110°, whereas collagen fibers orient between 0 and 180° in random devices (Supporting Figure 6B). These findings correlate with our findings that the collagen alignment coefficient is higher in the aligned device compared to the random device (Supporting Figure 6C).

Second, we quantified changes in tumor organoid response to matrix architecture in aligned and random devices under a low fluid flow. Tumor organoids began to spread in the direction of collagen fiber orientation within 24 h of culturing in aligned microfluidic devices (Supporting Figure 6A). Aspect ratio was significantly greater for tumor organoids cultured in aligned devices compared to random devices (Supporting Figure 6D). Also, tumor organoids were significantly less round in aligned devices compared with random devices (Supporting Figure 6F). Both aspect ratio and roundness parameters demonstrate tumor organoids changed their shape, such that they elongated in aligned devices. To understand what direction tumor organoids elongated, we measured the overall organoid orientation angle: tumor organoids cultured in aligned devices orient between 70 and 110°, which is in the direction of aligned collagen fibers, whereas tumor organoids cultured in random devices orient between 0 and 180° (Supporting Figure 6E). Further, we quantified the number and directionality of protrusions that developed in tumor organoids cultured in aligned and random devices. We did not observe significant differences between the total number of protrusions per organoid, but when we quantified the direction protrusions are pointing in, the majority of protrusions in aligned devices orient between 70 and 100°, which correlate directly to the orientation of aligned fibers (Supporting Figure 6G,H).

### 3.4 Chemokine Gradient Shape and Magnitude Dictates Directional Ruffling and Protrusions

**Figure 4:**
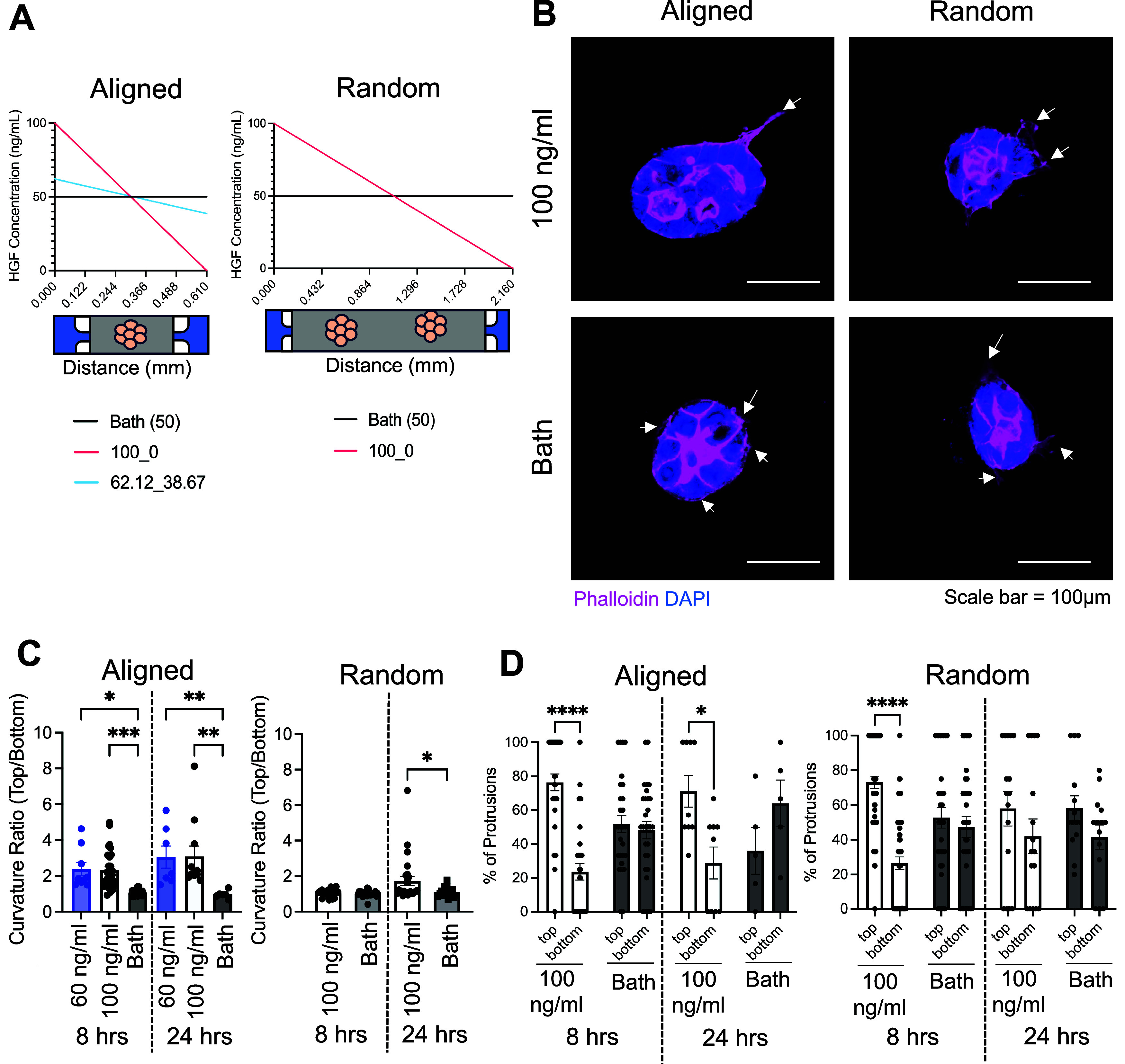
Biased ruffling and protrusions in MDCK cysts are dependent on chemokine gradient mean and magnitude (A) Schematic of the different HGF chemokine gradient conditions investigated. (B) Representative immunofluorescent images of MDCK cysts in aligned and random devices under HGF gradient (100 ng/mL) or bath after 8 h gradient exposure (phalloidin = pink, DAPI = blue, and scale bar = 100 μm). (C). Curvature ratio (top/bottom) (top/bottom of cyst) in aligned and random devices. (D). Percent protrusions in the top and bottom halves of each cyst. The top half is exposed to the larger concentration of the gradient. All data are presented as mean ± SEM; *n* = 3 replicates (16–50 cysts analyzed). For all experiments: ns = not significant, *p < 0.05, **p < 0.01, ***p < 0.001, AND ****p < 0.0001; ANOVA with Tukey’s posthoc analysis.

The distribution of HGF *in vivo* can be heterogeneous,^51−54^ suggesting the possibility that differential spatial gradients of HGF (magnitude and direction) can influence the initial steps required for epithelial morphogenesis, such as cell ruffling and protrusions.^55,56^ In our prior published studies in breast cancer collective migration, we demonstrated that there exist minimum and maximum mean and gradient magnitudes (slope) to promote directional tumor organoid migration.^39^ Thus, we asked if the mean and slope of the HGF gradient could affect the successful development of biased ruffles or protrusions. To test this, we exposed MDCK cysts to a set of different fixed linear gradients of HGF in both aligned and random devices (Figure 4A). In aligned devices, we tested two different gradient magnitudes (differences in concentration of HGF across the device divided by the total length of the device: slope =163.9 and 38.44 ng/mL/mm) and two different mean concentrations (50 ng/mL (Figure 4) and 25 ng/mL (Figure 3)). In random devices, we tested one set of gradient magnitude (slope = 46.29 ng/mL/mm) and mean concentration (50 ng/mL) in response to findings from the aligned devices (Figure 4A). For all studies, we analyzed cyst phenotype after 8 and 24 h of HGF gradient exposure. Specifically, we calculated the curvature (a measure of how much a curve deviates from a straight line) ratio of the top and bottom half of each cyst, with the top half exposed to the larger concentration of HGF. A larger curvature ratio demonstrates that there are more ruffles or protrusions in the top half of the cyst.

In aligned devices, we found that there existed a minimum mean concentration of HGF required for biased ruffles or protrusions to develop. A mean concentration of 25 ng/mL is sufficient to induce protrusions but not in a directional manner (Figure 3). Thus, the cysts exposed to mean concentration 25 ng/mL gradient conditions responded to both the mechanical architecture cue and aligned in the direction of the matrix with the overall cyst orientation in the direction of matrix architecture (Figure 3). When cysts were exposed to mean concentration 50 ng/mL gradient conditions, regardless of gradient magnitude (slope = 163.9 and 38.44 ng/mL/mm), after 8 h of gradient exposure, cysts developed biased ruffles or protrusions in the direction of the gradient that continued after 24 h (Figure 4B,C). In random devices, no differences were observed in curvature ratio after 8 h of gradient exposure, but after 24 h of gradient exposure, cysts began to develop biased ruffles or protrusions (Figure 4B,C). Our findings that cysts in random devices will take a longer time to respond to the gradients are expected because the random devices do not establish a gradient until 17 h after delivery (Figure 2).

We measured the percent of protrusions in the top and bottom half of each cyst. Similar to curvature ratios, there were significantly more cysts, with the majority of protrusions originating from the top half of aligned devices exposed to a 100 ng/mL gradient compared to the HGF bath (Figure 4D). These findings demonstrate that MDCK cysts respond to chemokine gradients of HGF in a directional manner, which could be used for inducing directional branching. From our experiments in Figures 3 and 4, we demonstrate the ability to promote directional branching in response to either matrix architecture or a chemokine gradient.

### 3.5 Cell Ruffling and Protrusions in Aligned Fibers Are Mediated by CDH3

**Figure 5:**
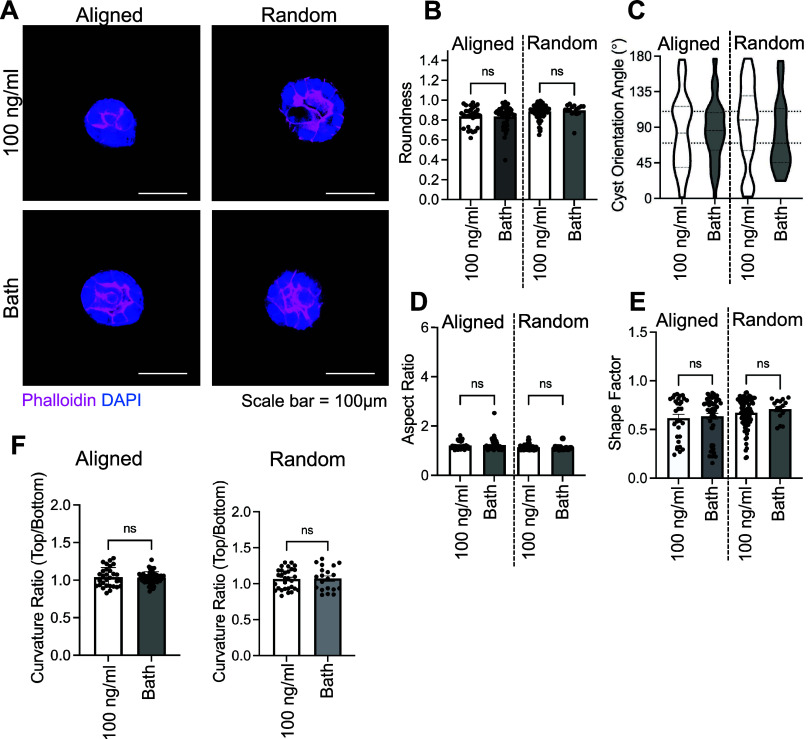
Cyst ruffling and protrusions are responsive to HGF and mechanical architecture *via* CDH3. (A) Representative immunofluorescent images of CDH3^−/−^ MDCK cysts in aligned and random devices under HGF gradient (100 ng/mL) or bath (phalloidin = pink, DAPI = blue, and scale bar = 100 μm). Cyst morphology: (B) roundness, (C) overall cyst orientation angle, (D) aspect ratio, (E) shape factor (measure of cell spreading), and (F) curvature ratio (top/bottom of cyst). All data are presented as mean ± SEM; *n* = 3 replicates (16–50 cysts analyzed). For all experiments: ns = not significant, *p < 0.05, **p < 0.01, ***p < 0.001, and ****p < 0.0001; ANOVA with Tukey’s posthoc analysis.

Next, we wanted to understand how MDCK cells sense and respond to fiber alignment. In prior work, our lab demonstrated that CDH3 has a functional role in inducing directional collective migration in tumor cells, and without CDH3, directional collective cell migration is inhibited.^57^ Thus, we asked whether CDH3 also affects the ability of MDCK cysts to respond to fiber alignment and form directional protrusions. To do this, we used CRISPR to knockout CDH3 in MDCK cells (CDH3^−/−^). After successfully knocking out CDH3, we created cysts, cultured in our microfluidic devices, and induced HGF gradient (100 ng/mL) for 8 h. CDH3^−/−^ MDCK cysts lost their ability to respond to the chemokine gradient and mechanical architecture (Figure 5A), as they remained round (Figure 5B) regardless of the conditions of the chemokine gradient or matrix alignment. CDH3^−/−^ cysts also did not have any significant differences in aspect ratio, a measure of the proportional relationship between the width and height of the cyst (Figure 5C). Also, CDH3^−/−^ cysts did not preferentially orient in any specific cluster angle orientation (Figure 5D), and there were no significant differences in shape factor, a measure of cell spreading, regardless of the device or gradient conditions (Figure 5E). Finally, there were no significant differences in curvature ratios (top/bottom) between the gradient and bath groups (Figure 5F). Altogether, our findings demonstrate that CDH3 was required for the development of ruffling and cell protrusions in response to HGF gradients and mechanical architecture.

## 4 Discussion

In this study, we created a set of microfluidic devices in which we can modulate chemokine gradients, matrix architecture, and interstitial fluid flow throughout the culture period to mimic dynamic culture conditions. First, we used a combination of computational and experimental methods to validate our microfluidic model. Second, we demonstrated the biological relevance of our microfluidics systems through investigating an *in vitro* model of epithelial morphogenesis and a collective migration model of tumor organoids. We successfully generated aligned and random matrix architectures through changing tissue geometries, and we could vary the mean and gradient magnitudes (slope) of chemokine gradients delivered to our model. Using the *in vitro* model of epithelial morphogenesis, we demonstrate that cyst ruffling and protrusions are dependent on both the chemokine gradient and matrix architecture. There exists minimum mean concentrations of chemokine gradient required to stimulate biased ruffling and protrusions. Finally, we demonstrate cyst ruffling and protrusions in response to chemokine gradient and matrix architecture are dependent on Cadherin-3 (CDH3).

While many studies investigate mechanical or chemical cues in microenvironments using *in vitro* platforms, the ability to study both in tandem has been limited. Other devices that couple mechanical and chemical cues within a microfluidic device include the generation of chemical concentration and substrate stiffness gradients^58^ or shear stress.^59−61^ A key advantage of our design is the ability to incorporate biochemical and biomechanical cues in a manner that allows us to probe the contributions of these two different cues separately, since the fiber alignment is in a perpendicular direction from the morphogen gradient formation. Additionally, our device is the first to our knowledge that incorporates the mechanical cue of fiber alignment with a morphogen gradient formation. This unique design allowed us to elucidate that both chemokine gradients and the matrix architecture can promote biased ruffling and protrusions. There exists a minimum threshold for the mean concentration that is required to stimulate initial ruffling steps of epithelial morphogenesis. Without reaching the minimum threshold mean concentration, cysts sense the surrounding matrix architecture and spread in the direction of the matrix fibers. Most studies of epithelial morphogenesis have only used chemical factors to induce cell protrusions that lead to branching.^26,30,32,33^ Here, we establish a set of threshold parameters for inducing biased cell ruffles and demonstrate the ability to promote directional cell protrusions in the direction of an aligned matrix. Our findings are consistent with biological processes, such as mammary duct morphogenesis^62,63^ and disease progression (*i.e.*, cancer),^64^ and provide a biologically relevant *in vitro* model to further investigate how chemical cues and matrix architecture initiate morphogenesis.

One potential limitation of our devices is that they have different length and width parameters, which could affect cell mechanosensing properties, especially cells close to the edge (*i.e.*, “edge effects”). To avoid any edge effects, we only analyze cysts that are in the center of our microfluidic devices. Further, many seminal papers, using computational and experimental methods, demonstrate the maximum distance cells can sense is on the subcellular scale (10–20 μm past the cell boundary).^65−69^ When cells are in the middle of each of our devices, there is at least a 20 μm distance between the cell boundary and the edge of our devices. This demonstrates the ability to use the devices to study cell response to independent and combinatory physiological cues.

In our previously published work, we cultured primary tumor organoids in static 3D-aligned collagen fiber hydrogels.^70^ In those culture systems, tumor organoids began responding to their surrounding matrix 48–72 h after exposure to aligned collagen fibers.^70^ Since tumor organoids respond to aligned collagen more quickly in our devices, which are under low levels of interstitial fluid flow, these findings suggest fluid flow may increase tumor organoids’ sensitivity to matrix architecture, resulting in cell elongation and protrusions within 24 h instead of 48–72 h of culture. Heightened cell sensitivity to fluid flow has been observed in other studies, as well. For example, culturing hepatocytes under fluid flow resulted in increased cell metabolic activity and drug sensitivity compared to static cultures.^71^ These studies, along with our current study, support the idea that fluid flow can activate cellular mechanisms to make cells more sensitive and responsive to the surrounding microenvironment.

To understand how MDCK cells respond to the aligned matrix, we performed knockout studies of CDH3, a known cell–cell adhesion marker that regulates collective invasion of breast tumor organoids.^57^ In our prior work, we demonstrated that CDH3 has a regulatory role in primary breast tumor organoids, which are epithelial-derived.^57^ When we knocked down CDH3 in primary breast tumor organoids, we observed a loss of collective migration potential. Primary tumor organoids without CDH3 could send out protrusions, but these protrusions did not adhere to the matrix to make a long-lasting protrusion and thus lost their ability to migrate collectively.^57^ These findings correlate with our current study: in CDH3^−/−^ cysts, we also did not observe any changes in cyst cell morphology parameters (roundness, aspect ratio, or orientation angle), with associated impaired cell spreading potential (shape factor). Together, these findings demonstrate that CDH3 regulates the ability of cells to make long-lasting extensions or protrusions that are essential for collective migration.

In this study, we do not investigate downstream signaling of CDH3, however, in our other work, we did link CDH3 function to known downstream regulators of migration in breast cancer animal models.^57^ Knocking down CDH3 in primary MMTV-PyMT tumor organoids inhibited laminin-332 and β-catenin associated signaling associated with loss of phospho-FAK signaling.^57^ Thus, in our prior work, we establish that CDH3 regulates downstream signaling pathways associated with FAK signaling. Since MMTV-PyMT tumor organoids are epithelial-derived, and MDCK cells are epithelial origin, we expect that CDH3 may also regulate laminin-332, β-catenin, and phospho-FAK-associated signaling. Our study establishes the foundation that CDH3 regulates MDCK cell sensing of chemokine gradients and matrix architecture and highlights the importance for future work to investigate downstream signaling mechanisms.

## 5 Conclusions

Results from our study demonstrate our ability to successfully control the matrix architecture, chemokine gradients, and interstitial fluid flow using a set of microfluidic devices. While we validate the biological relevance of our models using an *in vitro* model of epithelial morphogenesis, our devices have broad applications, including models for other complex tissues such as organized musculoskeletal tissues or models of various cancer tumor microenvironments. In addition to morphogens and growth factors, drugs can be introduced into the devices to see whether the cell response to them is altered by mechanical or chemical stimuli. Additional mechanical cues could be introduced by modulating the ECM hydrogel properties by increasing the stiffness or pore size. Our microfluidic device can be utilized in studies of cancer, developmental biology, mechanobiology, and high throughput drug screening.

## Data Availability

All relevant data are within the paper and its Supporting Information files.

## Supporting Information Available

Dimensions of aligned and random microfluidic devices (Supporting Figure 1); Additional fluid flow parameters for aligned and random microfluidic devices (Supporting Figure 2); Reynolds numbers and velocity values for specific locations within the tissue chamber for aligned and random microfluidic devices (Supporting Figure 3); FITC-dextran gradient establishment and maintenance for aligned and random devices at various time points (Supporting Figure 4); Verification of CDH3 knockout in MDCK cell line (Supporting Figure 5); Murine breast tumor organoids elongate and protrude in the direction of aligned fibers in a microfluidic device (Supporting Figure 6); MATLAB code used to quantify the number of protrusions and associated protrusion angles in MDCK cyst and MMTV-PyMT organoid analysis (PDF)
