# Supplementary material for: Using Microfluidics to Align Matrix Architecture and Generate Chemokine Gradients Promotes Directional Branching in a Model of Epithelial Morphogenesis
Source: ACS Biomater Sci Eng. 2024 Jul 15;10(8):4865–77. doi: 10.1021/acsbiomaterials.4c00245 (PMC11322918; doi:10.1021/acsbiomaterials.4c00245)
Supplement: Supplementary file 1 — ab4c00245_si_001.pdf [file ab4c00245_si_001.pdf]

Using microfluidics to align matrix architecture and generate chemokine gradients promotes directional branching in a model of epithelial morphogenesis

Jessanne Y. Lichtenberg[1*], Corinne E. Leonard[1*], Hazel R. Sterling[1], Valentina Santos Agreda[1], and Priscilla Y. Hwang[1,2]

[1]Department of Biomedical Engineering, Virginia Commonwealth University, Richmond, VA, USA, 23220

[2]Massey Comprehensive Cancer Center, Virginia Commonwealth University School of Medicine, Richmond, VA, USA, 23298

*These authors contributed equally to this work

Corresponding Author:

Priscilla Y. Hwang

hwangp2@vcu.edu

70 S Madison Street

Richmond VA 23220

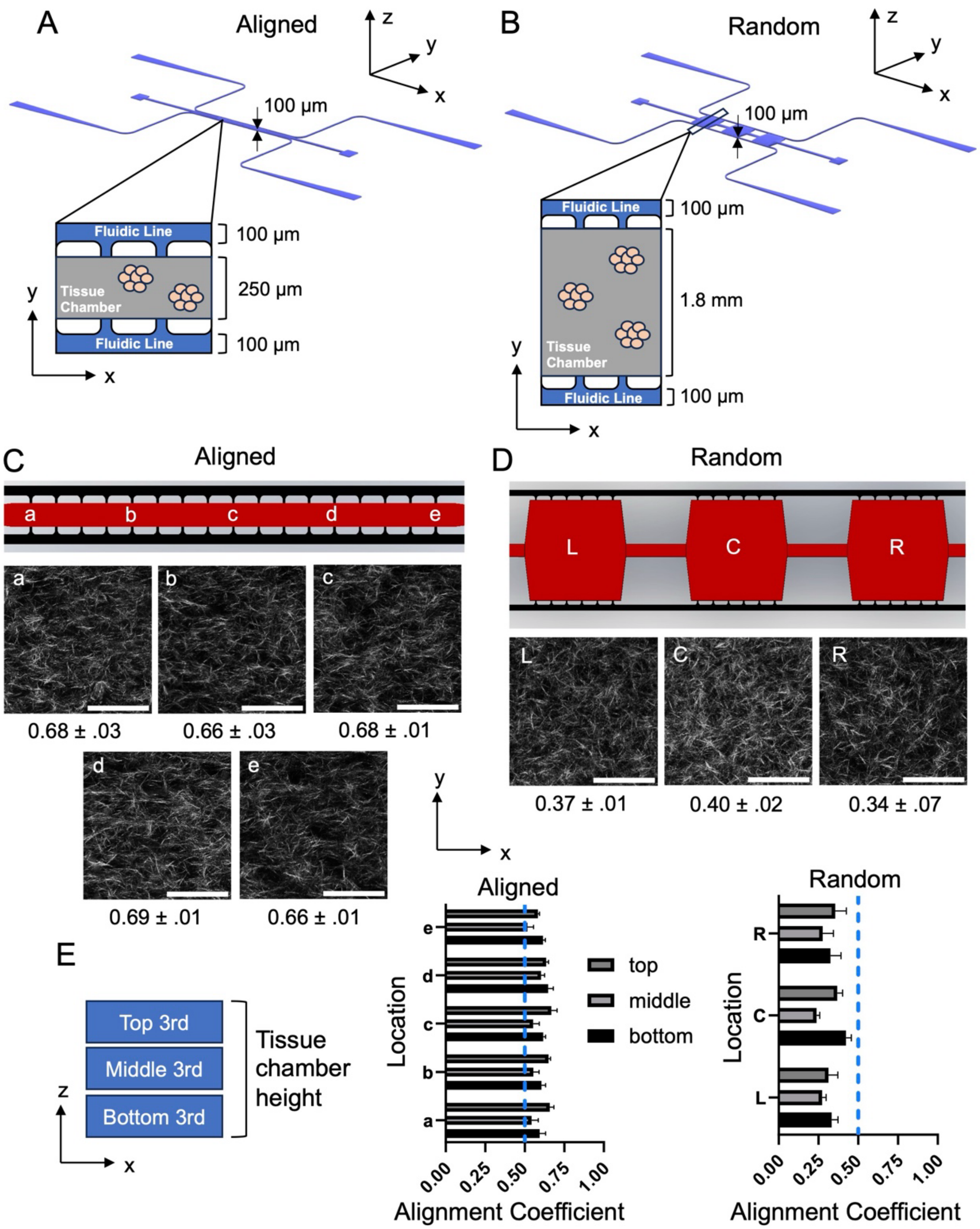

Supporting Figure 1: Dimensions of aligned and random microfluidic devices. (A) Aligned and (B) random device dimensions of tissue chambers and fluidic lines, depicting all channels as 100 μm in height. Representative images of collagen orientation with associated alignment coefficients at various locations within the tissue chamber of (C) aligned and (D) random device. (E) Alignment coefficients

representing top, middle, and bottom thirds of the z-direction within the tissue chambers at various locations within aligned and random devices. Blue dashed line represents the threshold of alignment (aligned, >0.5). n=4 devices. Data shown as mean ± SEM.

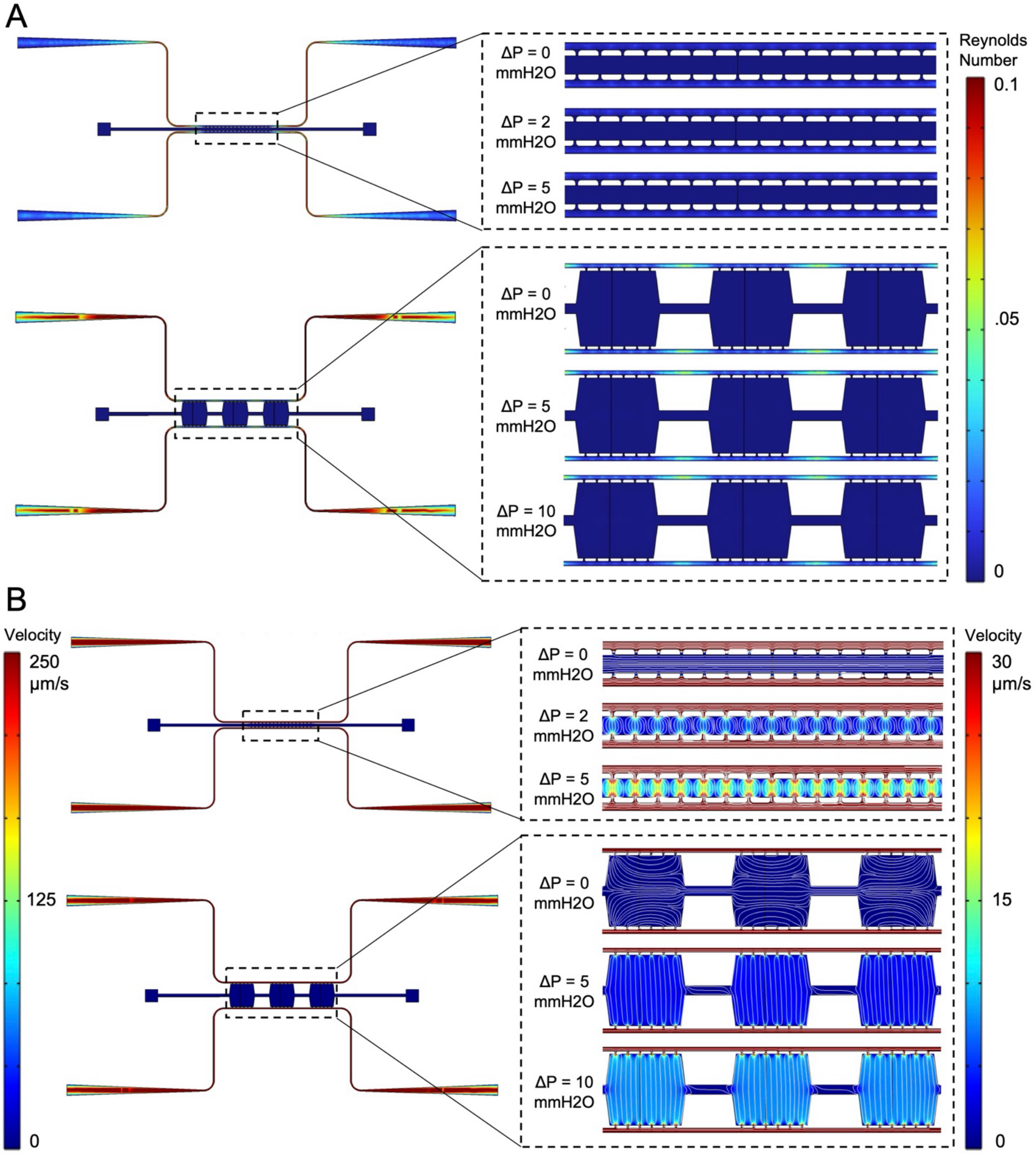

Supporting Figure 2: Additional fluid flow parameters for aligned and random microfluidic devices. (A) Reynolds number showing laminar flow ($Re < 1$) throughout both aligned and random devices at multiple pressure differences. (B) Velocity values and streamlines throughout aligned and random devices at multiple pressure differences.

A

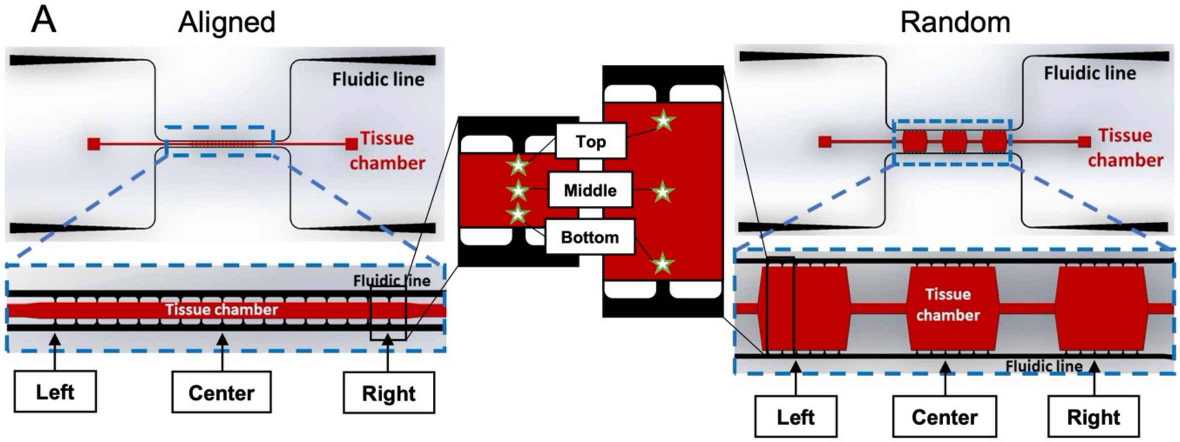

B

Reynolds Number

	Aligned				Random			
**Location**	**ΔP**	**Left**	**Center**	**Right**	**ΔP**	**Left**	**Center**	**Right**
**Top**	**ΔP = 0 mmH2O**	1.15E-6	2.37E-6	1.43E-6	**ΔP = 0 mmH2O**	4.54E-6	5.86E-6	5.04E-6
**Middle**		9.96E-7	3.40E-7	9.93E-7		3.22E-6	3.92E-6	3.45E-6
**Bottom**		1.15E-6	2.37E-6	1.43E-6		4.54E-6	5.86E-6	5.04E-6
**Top**	**ΔP = 2 mmH2O**	4.64E-5	4.54E-5	5.82E-5	**ΔP = 5 mmH2O**	1.05E-4	1.06E-4	1.02E-4
**Middle**		4.10E-5	3.94E-5	3.97E-5		6.74E-5	7.07E-5	6.77E-5
**Bottom**		4.64E-5	4.54E-5	5.82E-5		1.05E-4	1.06E-4	1.02E-4
**Top**	**ΔP = 5 mmH2O**	1.17E-4	1.13E-4	1.44E-4	**ΔP = 10 mmH2O**	2.11E-4	2.12E-4	2.04E-4
**Middle**		1.03E-4	9.83E-5	9.98E-5		1.35E-4	1.41E-4	1.35E-4
**Bottom**		1.17E-4	1.13E-4	1.44E-4		2.11E-4	2.12E-4	2.04E-4

C

Velocity (μm/s)

	Aligned				Random			
**Location**	**ΔP**	**Left**	**Center**	**Right**	**ΔP**	**Left**	**Center**	**Right**
**Top**	**ΔP = 0 mmH2O**	0.22	0.37	0.22	**ΔP = 0 mmH2O**	0.24	0.26	0.24
**Middle**		0.16	0.09	0.16		0.17	0.22	0.17
**Bottom**		0.23	0.37	0.23		0.24	0.26	0.24
**Top**	**ΔP = 2 mmH2O**	8.99	8.52	8.47	**ΔP = 5 mmH2O**	4.19	4.06	3.99
**Middle**		6.58	6.52	6.42		4.01	3.92	3.87
**Bottom**		9.17	9.06	9.24		4.08	4.00	3.99
**Top**	**ΔP = 5 mmH2O**	22.25	21.29	21.39	**ΔP = 10 mmH2O**	8.31	8.08	7.97
**Middle**		16.48	16.27	16.06		8.02	7.83	7.74
**Bottom**		23.17	22.62	22.84		8.21	8.02	7.97

Supporting Figure 3: Reynolds numbers and velocity values for specific locations within the tissue chamber for aligned and random microfluidic devices. (A) Key of locations measured (green stars)

within the tissue chamber of aligned and random device. (B) Measured Reynolds numbers and (C) velocity values (μm/s) at each location in the aligned and random device.

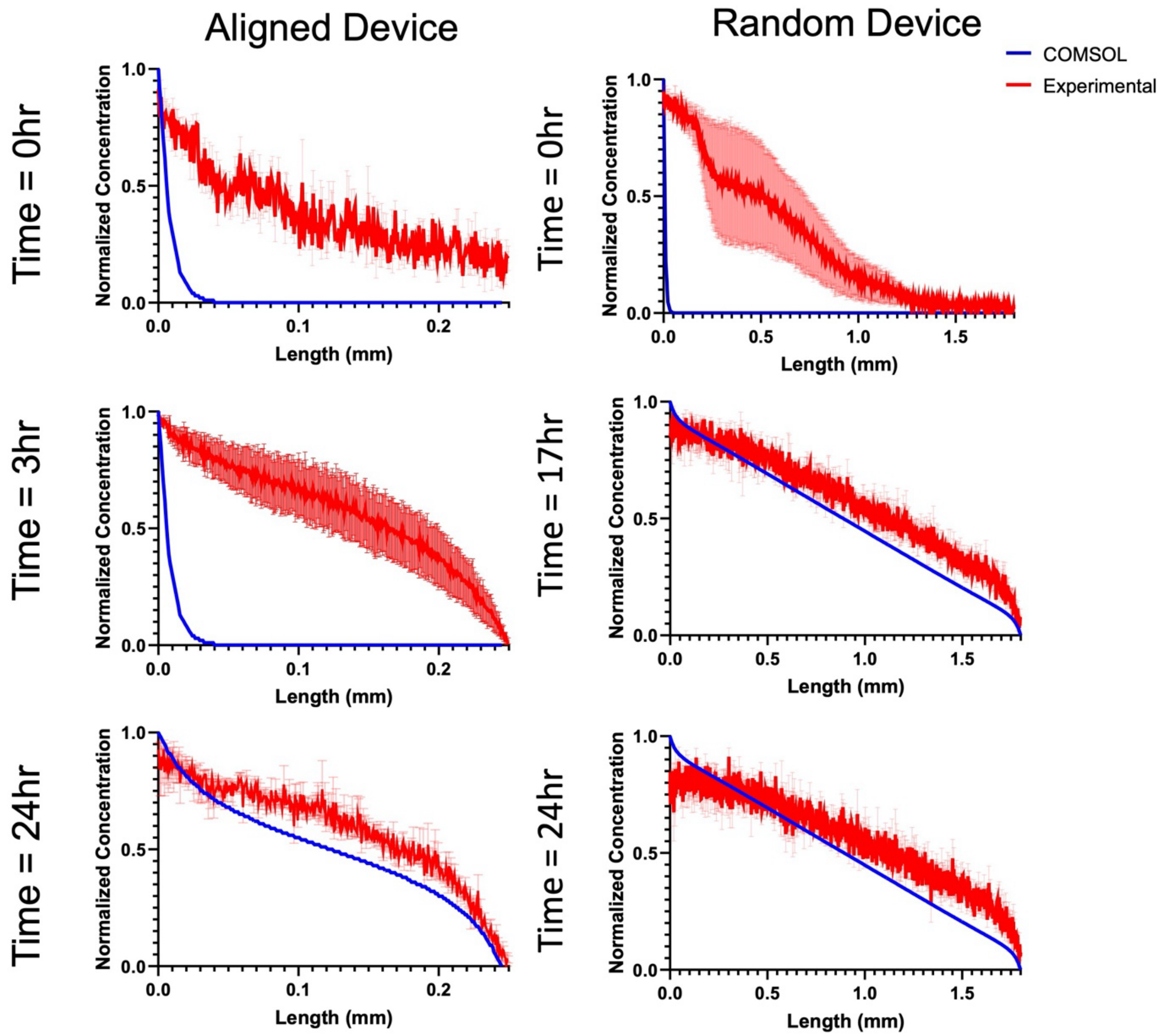

Supporting Figure 4: FITC-Dextran gradient establishment and maintenance for aligned and random devices at various time points. Data shown as mean ± SEM.

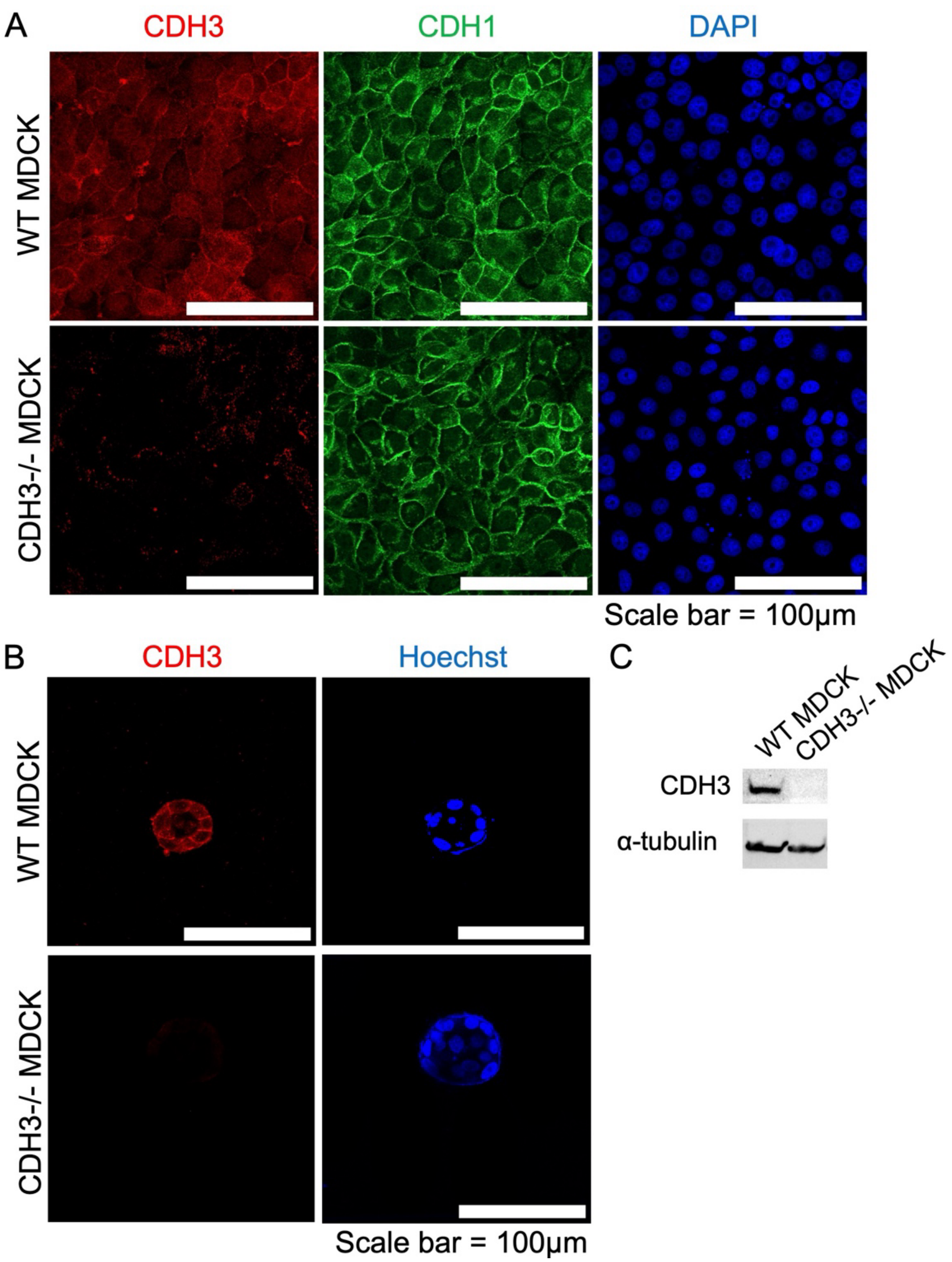

Supporting Figure 5: Verification of CDH3 knockout in MDCK cell line. (A) Immunofluorescence staining of wild type (WT) and cadherin-3 knockout (CDH3-/-) MDCK cells in 2D for CDH3 (red), CDH1 (green), and DAPI (blue). Scale bar = 100µm. (B) Immunofluorescence staining of WT and $CDH3^{-/-}$ MDCK cells in 3D cyst formation for CDH3 (red) and Hoechst (blue). Scale bar = 100µm. (C) Western blot for the WT and $CDH3^{-/-}$ MDCK cell lines.

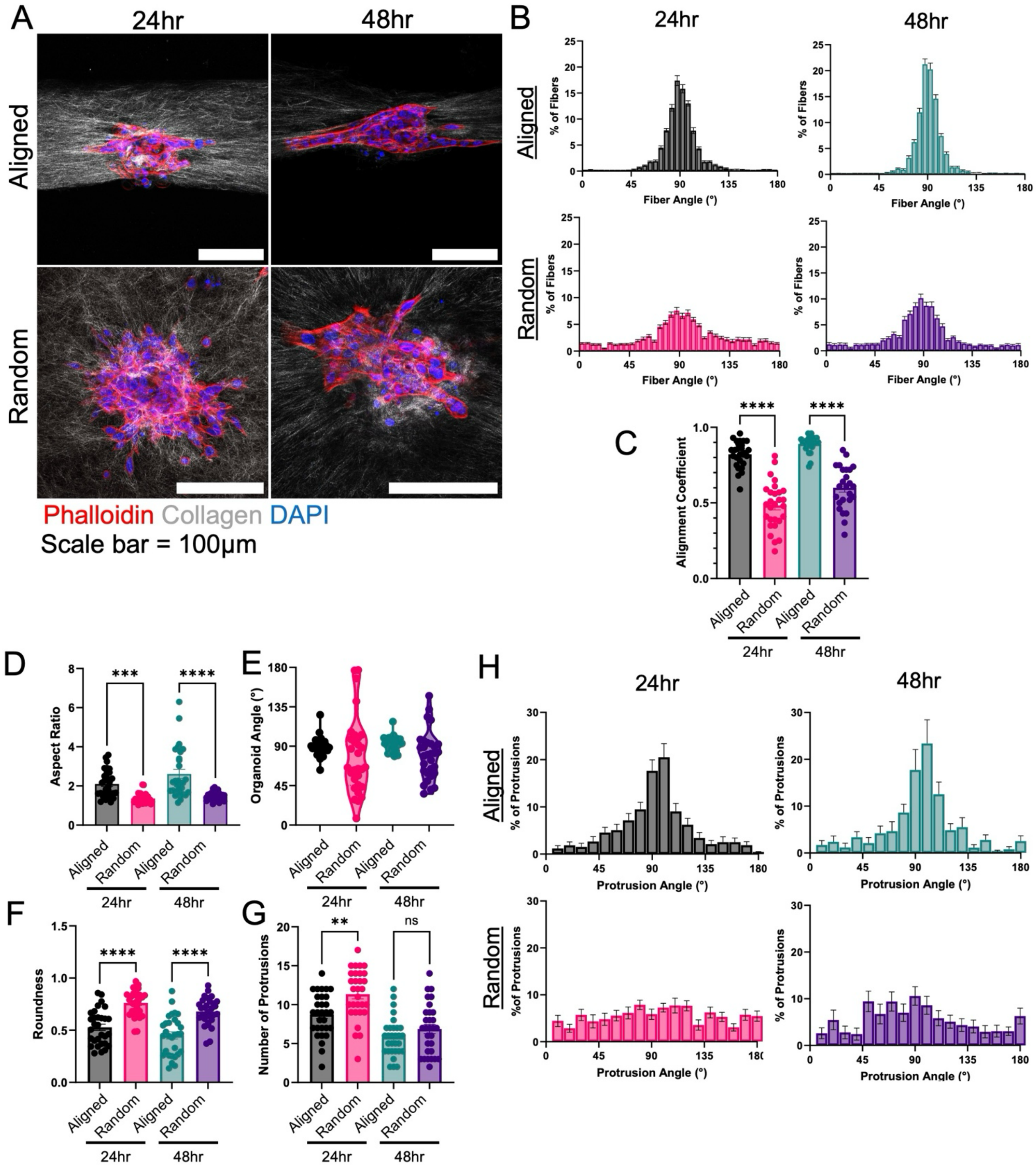

Supporting Figure 6: Murine breast tumor organoids elongate and protrude in direction of aligned fibers in microfluidic device. (A) Representative immunofluorescent images of tumor organoids from MMTV-PYMT mice in aligned and random devices after 24hr and 48hr. Scale bar = 100µm. (B)

Collagen fiber angle distributions. (C) Alignment coefficient of collagen fiber organization (aligned, >0.5). Characterization of organoid morphology: (D) aspect ratio, (E) organoid angle, (F) roundness, and (G) number of protrusions. (H) Protrusion angles of tumor organoids. Mean ± SEM. n=30 organoids from 3 mice. For all experiments: ns = not significant, *p<0.05, **p<0.01, ***p<0.001, ****p<0.0001; unpaired t-test.

Additional Supporting Information: MATLAB code used to quantify number of protrusions and associated protrusion angles in MDCK cyst and MMTV-PyMT organoid analysis. Briefly this code accepts immunofluorescent images or binary images of actin (phalloidin) and thresholds to find cell outline and centroid, then allows for user selection of protrusions to calculate associated angles and lengths of each protrusion.

Protrusion analysis

Corinne Leonard - Hwang Lab (June 1st, 2024)

```
close all
clear all
clc
```

Load image - tif format only, preferably IF actin image or something similar

```
z = imread(['insert_file_path']);
```

Generate Cell Outline

```
actinBW = im2gray(z);
actinBW = imadjust(actinBW);
fillBW = imfill(actinBW,"holes");
binActin = imbinarize(fillBW);
[cellOutline,L] = bwboundaries(binActin);
for i = 1:length(cellOutline)
    temp = cellOutline{i,1};
    s(i) = length(temp);
end
pos = find(s == max(s));
boundary = cellOutline{pos,1};
```

Find Centroid of Cell

```
stats = regionprops("table",binActin,"Centroid","Area");
a = stats.Area;
cell = find(a == max(a));
xCell = stats.Centroid(cell,1);
yCell = stats.Centroid(cell,2);
```

Plot Cell Outline on binary image

```
figure(1)
imshow(binActin)
hold on
for k = 1:length(boundary)
    plot(boundary(:,2), boundary(:,1), 'c', 'LineWidth', 2)
end
scatter(xCell,yCell,'filled','o','r')
hold off
```

Get protrusion points (user input)

```
i = 1;
imshow(~binActin);% display image (inverted to help with visualization)
hold on
title("click on each protrusion with mouse, press 'Enter' on keyboard when
finished")
while true
    try
    [x(i),y(i)] = ginput(1);
    scatter(x(i),y(i),'o','r') %plot points after clicked on
    i = i+1;
    catch
        break
    end
end
hold off
```

Plot line between protrusion and centroid

```
imshow(binActin)
hold on
scatter(xCell,yCell,'filled','o','r')
k = 1;
for k = 1:length(x)
    plot([xCell,x(k)],[yCell,y(k)])
    k = k+1;
end
```

Calculate the angle and distance from centroid (size) of each protrusion

```
angle = zeros(size(x));
dist = zeros(size(x));
vector = zeros(size(x));
```

```
origin = [1 0 0];
j = 1;


for j = 1:length(x)
    vectorTemp = [(x(j)-xCell) (y(j)-yCell) 0];
    if y(j) > yCell
        angle(j) = -
((atan2(norm(cross(origin,vectorTemp)),dot(origin,vectorTemp)))*(180/pi));
    else
        angle(j) =
(atan2(norm(cross(origin,vectorTemp)),dot(origin,vectorTemp)))*(180/pi);
    end
    dist(j) = sqrt((x(j)-xCell)^2 + (y(j)-yCell)^2); %in pixels
    j = j+1;
end
```

Output table

```
output = table(x',y',angle',dist');
output.Properties.VariableNames = ["x-location","y-
location","Angle(o)","Length(px)"];
output
output1 = [angle', dist'];
output1
```
